# Equity in medical school interviews: a cross-sectional analysis of school type, gender, and widening participation status

**DOI:** 10.1186/s12909-026-09790-8

**Published:** 2026-07-07

**Authors:** Umika Moorjani, Celia A. Brown, Lidia Bonomi, Amir H. Sam, Shahid A. Khan

**Affiliations:** Imperial College School of Medicine, Imperial College, London, UK

**Keywords:** Medical school admissions, Multiple mini-interviews, Widening participation, Socioeconomic status, Educational equality, Gender, School selectivity

## Abstract

**Background:**

Multiple Mini Interviews (MMIs) are widely used in medical school admissions to assess non-academic attributes. While MMIs are designed to promote fairness, questions remain about how applicant background may influence performance. In 2015, a single-institution UK study found no significant impact of school background or socioeconomic status on MMI scores. A decade later, this study re-examines those associations in a similar UK context.

**Methods:**

In this cross-sectional observational study, we analysed data from 545 home applicants to Imperial College School of Medicine during the 2025 admissions cycle. Using multiple linear regression, we explored the relationship between total MMI score and applicant gender, school type, and widening participation (WP) status. Station-level regressions were also conducted.

**Results:**

The regression model was statistically significant and explained 7.6% of total score variance. Out of a maximum score of 70, female applicants scored on average 3.2 points higher than males (95% CI: 2.1 to 4.2, *p* < 0.001). Applicants from independent and state-selective schools scored 2.4 (95% CI: 1.0 to 3.9, *p* = 0.001) and 1.3 points higher (95% CI: 0.0 to 2.7, *p* = 0.046), respectively, than state non-selective peers. WP status was not a significant predictor. Station-level analyses revealed no consistent disadvantage for WP applicants.

**Conclusion:**

Our findings suggest that, unlike earlier UK research, school background and gender now show modest associations with MMI performance. WP applicants, however, perform comparably to their peers, indicating progress towards equitable access. Future work should explore the potential impact of coaching and familiarity with interview formats to inform MMI design.

## Background

Multiple Mini Interviews (MMIs) offer a structured, station-based format to assess a broad range of non-academic attributes such as communication skills, ethical reasoning, empathy, and professionalism [1–3]. Compared to traditional panel interviews, MMIs are considered to reduce individual interviewer bias by involving multiple assessors across a range of short, standardised scenarios [4]. Their format is also associated with improved reliability and predictive validity, especially in assessing interpersonal skills [5]. As such, MMIs have been widely adopted to aid selection in medical school admissions processes across the globe [6–8].

Despite these strengths, there is growing evidence to suggest MMIs may not be entirely immune to the influence of applicants’ demographic or educational background. Various studies have found associations between MMI performance and characteristics such as gender, socioeconomic status (SES), and school type - raising important questions about the equity of selection processes. An earlier study evaluating the influence of socioeconomic status on MMI performance in a UK medical school setting, suggested no statistically significant differences based on SES, parental education, or school background [9]. These observations led to conclusions that MMIs did not disadvantage applicants from underrepresented or lower-income backgrounds [9].

However, subsequent research across multiple countries has yielded mixed findings. Leduc et al. [10], analysing MMI performance in over 1,000 applicants to Canadian medical schools, found that applicants who were female, older, or from higher-income households scored significantly better- while those with Asian-born parents tended to score lower. Knorr et al. [11] observed that younger female candidates outperformed their male peers in both interview and simulation stations, although gender differences diminished with age. In America, Jerant et al. [12] reported that MMI scores were lower on average for applicants from lower SES backgrounds and certain ethnic minority groups, while Henderson et al. [13] found institutional variation in the impact of background factors on both MMI and traditional interview performance.

These findings may be somewhat explained by increased candidate familiarity with interview formats, as demonstrated by Myung et al. [14] in Korean MMIs. Abdulghani et al. [15] similarly highlighted that cultural and language factors may introduce subtle disadvantages in medical school interviews. These findings collectively suggest that, although MMIs offer advantages in structure and standardisation, their fairness in practice may be influenced by broader contextual and structural factors—such as educational opportunity, cultural capital, and access to preparatory resources.

Within the UK context, these concerns are reflected by widening participation (WP) initiatives, which aim to address structural inequalities in access to medical education in line with national policy priorities to improve healthcare workforce diversity and social mobility [16]. However, WP is not a singular or uniformly defined construct across UK medical schools and is typically operationalised using composite indicators incorporating socioeconomic measures, educational context, and area-based deprivation metrics. While some indicators, such as eligibility for free school meals, are generally regarded as relatively robust, others, such as area-based measures, may have limited individual-level specificity and a higher potential for misclassification [17, 18]. The lack of a standardised national definition of WP presents a challenge for research in this area by potentially influencing observed associations between WP status and admissions outcomes, including MMI performance [16].

Against this backdrop of evolving policy priorities and methodological complexity, our study builds on the 2015 UK analysis of Taylor et al. [9], applying similar methods a decade later within a contemporary, remotely-delivered MMI model to explore whether patterns have changed, particularly with respect to school background, gender, and widening participation status. In doing so, we aimed to contribute to the growing body of work examining how medical school selection tools can support equity in access and uphold principles of fairness, diversity, and inclusion. This question is particularly timely given the recent shift toward virtual or hybrid MMIs in the UK post-COVID, alongside ongoing efforts by medical schools to refine widening participation initiatives and contextualised admissions criteria. We therefore aimed to evaluate whether variation exists in MMI outcomes across demographic groups. Such analysis supports efforts to ensure that selection processes are equitable and inclusive and helps to identify whether any unintentional barriers exist for applicants from different educational or social backgrounds.

## Methods

### Study design and aim

This was a retrospective cross-sectional study using routinely collected admissions data from home (UK fee-paying) applicants to Imperial College School of Medicine during the 2025 admissions cycle. The aim was to examine associations between applicant background characteristics - specifically gender, school type (state non-selective, state selective, or independent), and widening participation (WP) status - and performance in Multiple Mini Interviews (MMIs). The primary outcome was total MMI score (out of 70, based on a maximum score of 10 in each of seven separate MMI stations). Multiple linear regression was used to assess associations between total MMI score and the independent variables. Station-level regressions were also conducted as secondary analyses.

### Participants and data sources

The dataset included all home (UK fee-paying) applicants interviewed during the 2025 admissions cycle who had complete data for MMI scores and demographic variables. This restriction to home applicants aimed to minimise the potentially confounding impact of differing educational systems and national contexts associated with international applicants. All 568 home fee-paying applicants invited to interview in 2025 completed the full MMI process, comprising all seven stations.

Of the 568 interviewed applicants, 23 were excluded from analysis solely due to incomplete demographic or school-type data, resulting in a final analytical sample of 545 applicants.

Demographic data, including gender and WP status, were based on UCAS application data and institutional records. All participants included in the analysis had self-identified as male or female; no applicants reported a non-binary gender identity. WP classification followed Imperial College’s criteria (Appendix 1).

Names of schools attended at the time of UCAS application (A-levels or equivalent examination period) were included in the dataset and independently reviewed for categorisation. School type was classified into three categories: state non-selective, state selective, and independent, using publicly available data from UK government websites (e.g. UK.gov, Get Information About Schools, Ofsted) and cross-referenced with each school’s own current website. Classification was performed manually and independently by the study team.

### MMI format and scoring

All applicants completed a structured Multiple Mini Interview (MMI) comprising seven 5-minute stations designed to assess key non-academic attributes such as communication, professionalism, and ethical reasoning. The MMI was conducted entirely remotely as a single, integrated interview process, consisting of three asynchronous stations followed at a later date by four live online stations. All applicants were required to complete all seven stations, and all stations contributed equally to the final MMI score. There was no selection or progression step between stations; applicants were not screened or excluded between asynchronous and live components of the interview.

For asynchronous stations, candidates accessed a secure online platform via an individualised link and were presented with a novel scenario at the time of access; questions were not provided in advance. Candidates were required to record and upload a single response immediately within a fixed time limit, and recordings could not be edited or re-submitted. A system test run was undertaken prior to the interview cycle, and no significant technical issues affecting performance were identified. All stations, including the live stations, were conducted online.

Each station was scored out of 10, generating a total score out of 70, which served as the primary outcome variable in the analysis. For all stations, scores were awarded using a structured marking scheme comprising up to 7 marks for content and up to 3 marks for communication skills. Offer status was not analysed due to the high offer rate among home applicants (> 89%), which limited statistical power. Therefore, the analysis focused on overall MMI performance and individual station scores as continuous outcomes.

Each station was scored independently by a single interviewer. A total of 128 interviewers participated in the 2025 cycle, typically senior clinicians (Specialty Trainee Year 3 and above), the majority of whom had prior MMI experience. A summary of the professional roles represented within the interviewer cohort is shown in Table 1.

**Table 1:** Interviewer professional roles (*N* = 128)

Academic Staff	14 (10.9%)
Clinical Academic	1 (0.8%)
Clinical Teaching Fellow	4 (3.1%)
Consultant	39 (30.5%)
General Practitioner	49 (38.3%)
Senior Clinical Fellow	1 (0.8%)
Specialty Doctor	4 (3.1%)
Teaching Fellow	16 (12.5%)
Total	128

Interviewers were required to complete structured training before participating to promote consistency and reduce inter-rater variability. This included an electronic MMI marking module outlining station-specific indicators and scoring guidance. For each station, interviewers were provided with explicit positive and negative behavioural and content indicators (e.g. demonstration of empathy, awareness of specific factors, or failure to offer appropriate support) to guide scoring decisions. Interviewers were also required to complete Equality, Diversity and Inclusion training and sign a confidentiality agreement.

### Statistical analysis

A power calculation was conducted prior to analysis. Assuming a medium effect size (Cohen’s f² = 0.15), four predictors, an alpha level of 0.05, and 90% power, the minimum required sample size was 108 participants. With a final sample of 545, the study was well-powered to detect small to medium effects.

Statistical analysis was performed in Microsoft Excel using raw, untransformed data tables. Multiple linear regression was used to assess associations between total MMI score and predictors: gender, WP status and school type. School type was treated as a categorical variable with state non-selective as the reference group.

Subgroup analyses were conducted for each individual MMI station score. To account for multiple comparisons across the seven station-level regression models, a Bonferroni-corrected significance threshold of *p* < 0.0071was applied.

## Results

### Participant characteristics

A total of 545 home applicants to Imperial College School of Medicine were included in the analysis (Table 2).

**Table 2:** Participant characteristics with MMI scores

Category	*N*	%	Mean MMI Score	SD
Total	545	100.0%	50.7	6.3
**Gender**				
Female	243	44.6%	52.5	5.5
Male	302	55.4%	49.3	6.6
**Widening Participation**				
WP applicants	179	32.8%	50.9	6.8
Non-WP applicants	366	67.2%	50.6	6.1
**School Type**				
State non-selective	118	21.7%	49.4	6.5
State selective	272	49.9%	50.7	6.4
Independent	155	28.4%	51.8	5.8

Descriptive statistics for total and station-level MMI scores are shown in Table 3.

**Table 3:** Distribution of total and station-level MMI scores (*N* = 545)

Assessment component	Mean (SD)	Median (IQR)	Minimum score	Maximum score
Total MMI score (out of 70)	50.7 (6.3)	51 (47–55)	28	66
Station 1 (Async – teamwork & leadership)	6.84 (1.36)	7 (6–8)	2	10
Station 2 (Async – communication skills)	7.07 (1.32)	7 (6–8)	3	10
Station 3 (Async – candidate-focused questions)	7.21 (1.47)	7 (6–8)	3	10
Station 4 (Live – ethics)	7.50 (1.63)	8 (6–9)	2	10
Station 5 (Live – motivation for medicine)	7.47 (1.73)	8 (6–9)	3	10
Station 6 (Live – interpersonal characteristics)	7.24 (1.51)	7 (6–8)	3	10
Station 7 (Live – ethics)	7.34 (1.43)	7 (7–8)	3	10

*SD* standard deviation, *IQR* interquartile range

### Total MMI score regression

A multiple linear regression model (Table 4) examined the association between applicant characteristics and total MMI score (out of 70), with predictors being school type, gender, and WP status.

**Table 4:** Multiple linear regression for total MMI scores

Candidate Variable	Coefficient (B)	95% CI	*p*-value
Independent school*	+ 2.43	[0.95, 3.92]	0.001
State selective school*	+ 1.34	[0.02, 2.66]	0.046
Female ( vs. Male)	+ 3.17	[2.14, 4.20]	< 0.001
WP status (Yes vs. No)	+ 0.68	[-0.42, 1.79]	0.230

*vs state non selective school

As shown in Table 4, applicants from independent schools scored on average 2.43 points higher than those from non-selective state schools (3.5%, *p* = 0.001), while those from selective state schools scored 1.34 points higher (1.9%, *p* = 0.046). Female applicants outperformed male applicants by 3.17 points on average (4.5%, *p* < 0.001). WP status was not a significant predictor (+ 0.68 points, 0.97%, *p* = 0.23). The model explained approximately 7.6% of the variance in total scores (adjusted R² = 0.076).

### Station-level analyses

**Fig. 1:**
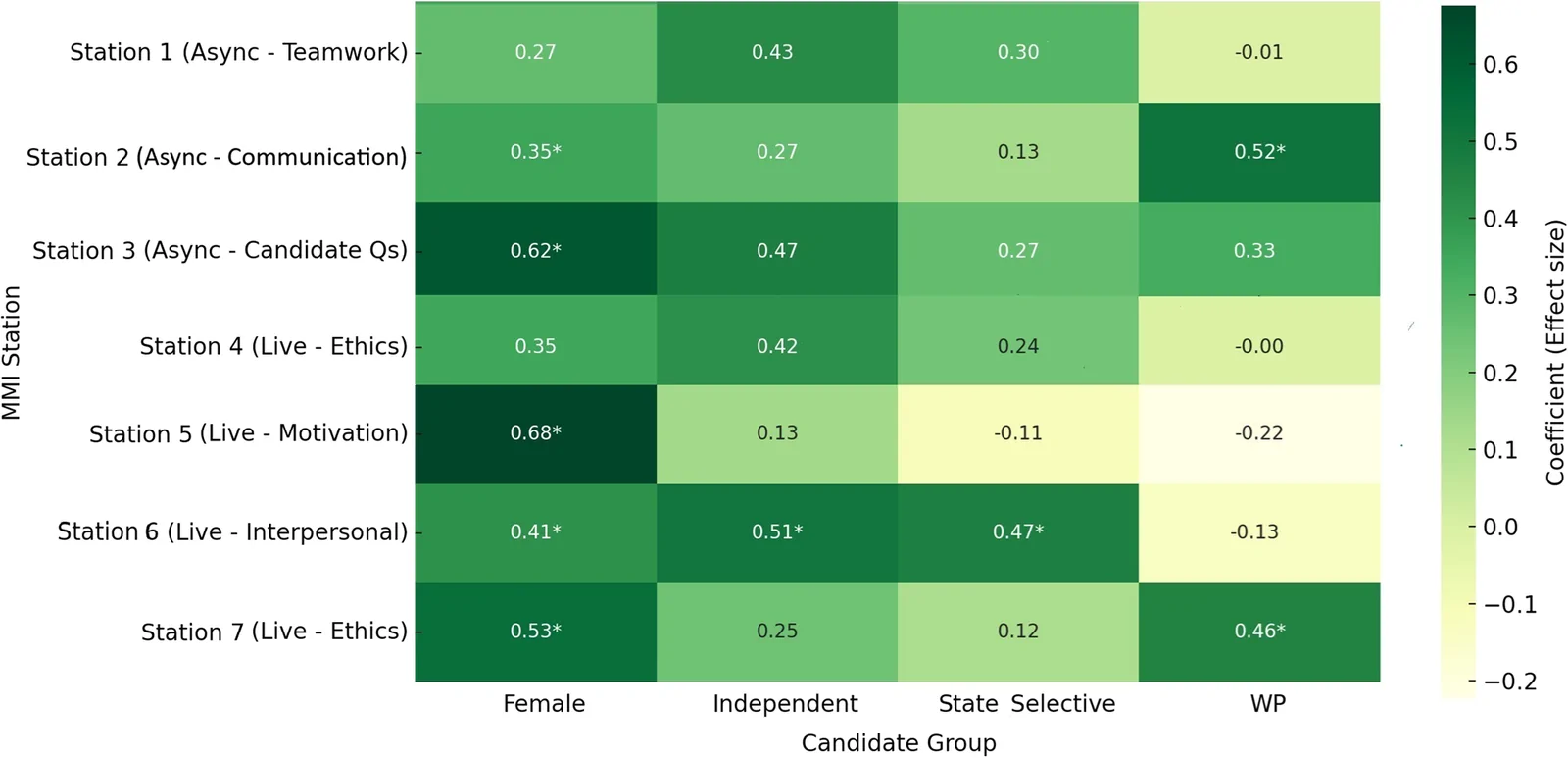
Heatmap of Interview score effects by group and station (* *p* < 0.0071)

Separate regression models were also run for each of the seven individual MMI stations using the same variables as in the total score model—school type (with state non-selective as the reference), gender, and WP status. Full regression outcomes for each demographic variable per station are presented in Table 5. Figure 1 provides a visual summary of the effects of each demographic variable by station, with statistically significant results marked.

**Table 5:** Multiple linear regression for individual station scores

MMI Station	Predictor	Coefficient (B)	t-Stat	*P*-value	*R* ^2^
Station 1 (Asynchronous - Teamwork & leadership)	Independent school	0.432	2.561	0.011	0.023
	State selective school	0.299	1.990	0.047	
	Female	0.267	2.290	0.022	
	WP background	-0.010	-0.080	0.936	
Station 2 (Asynchronous – Communications skills)	Independent school	0.270	1.678	0.094	0.052
	State selective school	0.129	0.898	0.369	
	Female	0.354	3.186	0.002	
	WP background	0.516	4.299	< 0.001	
Station 3 (Asynchronous - Candidate focused questions)	Independent school	0.472	2.659	0.008	0.065
	State selective school	0.267	1.690	0.092	
	Female	0.619	5.046	< 0.001	
	WP background	0.332	2.503	0.013	
Station 4 (Live - Ethics)	Independent school	0.418	2.068	0.039	0.020
	State selective school	0.235	1.308	0.192	
	Female	0.352	2.518	0.012	
	WP background	-0.004	-0.029	0.977	
Station 5 (Live - motivation for medicine)	Independent school	0.132	0.624	0.533	0.047
	State selective school	-0.112	-0.597	0.551	
	Female	0.676	4.624	< 0.001	
	WP background	-0.223	-1.415	0.158	
Station 6 (Live - interpersonal characteristics)	Independent school	0.509	2.742	0.006	0.040
	State selective	0.471	2.855	0.004	
	Female	0.413	3.217	0.001	
	WP background	-0.129	-0.933	0.351	
Station 7 (Live - Ethics)	Independent school	0.247	1.417	0.157	0.057
	State selective school	0.115	0.743	0.458	
	Female	0.534	4.433	< 0.001	
	WP background	0.456	3.503	< 0.001	

Candidates from state selective and independent schools demonstrated slightly higher scores on specific stations compared to those from state non-selective schools. Female gender was associated with higher scores across most stations. After applying a Bonferroni correction for multiple comparisons, no station showed a disadvantage for WP applicants; in fact, some stations showed a positive association between WP status and performance. The variance explained in individual station scores was always less than 10%.

## Discussion

This study explored whether gender, school type, or widening participation (WP) status influenced performance in MMIs among UK medical school applicants. Using data from 545 applicants to Imperial in 2025, we found modest but statistically significant differences in total MMI scores linked to gender and school background. Female applicants and those from independent or selective state schools achieved slightly higher scores overall, while WP status showed a small positive association in some stations with no overall effect on total score.

These findings provide a noteworthy counterpoint to the conclusions of Taylor et al. (2015) [9], whose study suggested that MMIs did not disadvantage applicants based on school type or socioeconomic status. Although differences in MMI delivery should be acknowledged, particularly the use of a fully remote format incorporating both live and asynchronous stations in the present study, the comparison remains informative in highlighting how patterns of equity in MMI performance may have evolved over time. Our results were consistent with the findings of Taylor et al. regarding WP applicants, showing no evidence of disadvantage for this group [9]. A decade later, our results suggest that school background may now be modestly associated with MMI performance. Taken together, these findings suggest that while the mode of MMI delivery in our study differed to that of Taylor et al., questions of equity remain relevant and warrant continued evaluation within contemporary selection contexts. This shift raises important questions about how preparation for MMIs has evolved and whether certain school environments have become better positioned to support applicants in navigating these interview formats.

The small but consistent advantage for applicants from independent schools suggests that familiarity with MMI-style tasks, access to coaching, or confidence in high-pressure interactions could still confer benefits. We hypothesise that over time, certain schools may have adapted more effectively to the evolving demands of medical school selection, including the growing emphasis on MMIs and the incorporation of virtual interview formats.

Female applicants scoring higher in MMIs is in keeping with prior international studies. Knorr et al. (2019) [11], examining German MMI data, found younger women performed better in interview and simulation stations. While cultural and educational contexts differ, these findings support the idea that communication skills - central to MMI performance - may differ by gender during early adulthood. This observed difference could perhaps reflect socialisation patterns, confidence, or familiarity with verbal performance tasks.

Our findings regarding WP status are encouraging. The lack of performance disadvantage suggests that WP initiatives, such as tailored applicant support, may be achieving their intended aims. These results align with literature indicating that structured, multi-station interviews can reduce bias and level the playing field for underrepresented applicants [5, 9, 12]. However, WP represents a heterogeneous construct, and the interpretation of WP-related findings should account for variation in how WP is operationalised across medical schools. Differences in WP definitions may contribute to heterogeneity in observed associations with MMI performance, which has implications for the comparability and transferability of findings across institutions.

Importantly, equity in interview performance must be interpreted within the broader admissions pathway. Although the MMI itself did not appear to disadvantage WP applicants or those from non-selective state schools, this does not address whether applicants from these groups were proportionately represented among those invited to interview. A fair interview process alone cannot fully mitigate inequities that may arise earlier in selection. As this study examined performance among applicants who progressed to interview, the findings should be interpreted within the context of a pre-selected cohort and do not necessarily reflect representation or equity at earlier stages of the admissions process.

It is also notable that, despite comprehensive interviewer training and standardisation efforts, 7.6% of score variance was explained by background characteristics. Although modest, this suggests that interview performance may still be influenced by factors beyond the intended constructs assessed. The relatively narrow interquartile ranges across stations indicate clustering of scores within a highly competitive cohort, which may limit variability and the magnitude of detectable effects; findings should therefore be interpreted cautiously. As others have argued, MMIs - though structured - are not entirely immune to broader sociocultural influences [13, 15]. Although not necessarily modifiable, identifying preparation resources or educational experiences that confer advantage will be important in refining admissions design to promote fairness [19].

### Limitations and generalisability

This study is based on data from a single admissions cycle at a single UK medical school, which may limit the generalisability of findings to other institutions or selection contexts. While key demographic characteristics were included in the analysis, data on other potentially relevant factors- such as ethnicity, first language, or neurodiversity- were not available and therefore not accounted for. In addition, the categorisation of school type was based on publicly available data and manual classification, which may introduce some inaccuracies; this method may not reflect recent changes in school policies or within-school variation. It is also possible that some applicants changed schools between GCSEs and A-levels, limiting the extent to which a single school classification accurately reflects educational background or its impact.

As discussed, WP status was treated as a single composite variable in line with institutional practice, which may mask heterogeneity within WP groups. This limits assessment of individual indicators and may reduce the generalisability of WP-related findings to institutions using alternative WP definitions.

A further limitation is the high post-interview offer rate (approximately 89%), reflecting a greater degree of pre-interview screening. While analysis of score variation still provides insight into equity within the interview process itself, potential inequities may be more pronounced earlier in the admissions pathway. This study did not examine applicant characteristics at the point of application or shortlisting, and therefore cannot assess whether underrepresented groups were differentially filtered out prior to interview. Accordingly, conclusions relate specifically to performance within the interview stage and should not be extrapolated to the full applicant cohort.

Additionally, despite standardised training and calibration, some variability in scoring may persist due to the heterogeneity of the interviewer cohort. The relatively restricted spread of station scores may also reflect structured marking and assessor calibration within a high-performing cohort, which may reduce variability and constrain effect sizes.

Finally, since these MMI stations were conducted virtually, and some stations asynchronously, this may have influenced applicant performance in ways that differ from fully live, in-person interview formats. Further multi-centre studies would be valuable to confirm whether these patterns persist across settings.

### Future directions

This study contributes to the growing body of research exploring equity in medical school admissions. As Imperial, along with other institutions, transitions back to a fully live MMI format post-pandemic, it will be important to re-evaluate whether similar patterns of performance persist in face-to-face settings. Further research could also explore additional applicant characteristics such as ethnicity, neurodiversity, and access to interview preparation resources. The modest advantage observed among independent school applicants raises the possibility that certain schools may have developed more effective strategies for preparing students for the MMI format over time. Future MMI development should consider reducing reliance on questions where rehearsed or coached responses may offer undue advantage.

Finally, while this analysis focused on interview performance at the point of selection, it would be valuable to explore the predictive validity of MMIs in future studies to assess how well they identify candidates who go on to succeed in their medical school training. It would also be useful to examine equity across the full admissions pathway, including pre-interview shortlisting and screening processes, to better understand where differential attainment may arise.

## Data Availability

The datasets generated during this study are not publicly available due to institutional policy but are available from the corresponding author on reasonable request for researchers who meet the criteria for access to confidential data.

## Appendix 1

### Criteria for contextual admissions at Imperial College London^1^

If you are a student with Home fee status or an appropriate UK residential status, you may be considered through our contextual admissions route if one of the following also applies to you:

- You are a care leaver or have spent time in care under a local authority, or
- Your home address* postcode falls into England’s index of multiple deprivation quintile one, or
- You currently receive UK government funded free school meals (FSM), or have received them during the last six years up until the end of your final year at school or college; or
- Your home address* postcode falls into POLAR4 quintile 1 or 2 and you also meet one of the following criteria:
  - ⚬ You are the first in your family to attend university
  - ⚬ You attend an English school where the key-stage 5 performance is below Imperial's percentile threshold of 20%
  - ⚬ You attend a school in England, Scotland, Wales or Northern Ireland where the Free School Meals percentile is above Imperial's percentile threshold of 80%

*Your home address is your place of permanent residence and not your school or boarding school.
